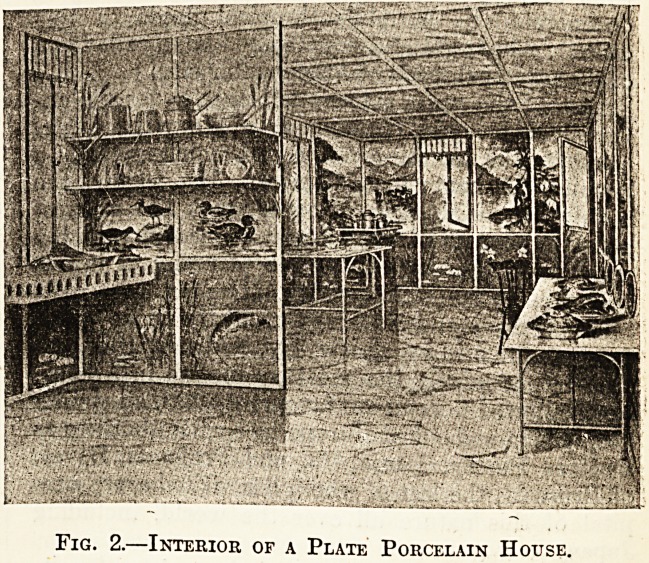# A New Building Material.—Plate Porcelain

**Published:** 1912-02-17

**Authors:** 


### HOSPITAL ARCHITECTURE AND CONSTRUCTION.

**[Communications on this subject should be marked "Architecture" in the left-hand top corner of the envelope.]**

### A New Building Material.—Plate Porcelain.

The architect of last century was a very conservative gentleman, and approached new materials with diffidence; the architect of the twentieth century cannot afford to ignore the progress of science in the substitutes which are daily brought to his notice, as, for example, modern Doulton ware instead of stone for wall facings, or asbestos

Fig 1.—Exterior of a Plate Porcelain House.

roofing tiles instead of slates. The former is more adaptable to monolithic concrete construction, and the latter is a better non-conductor of heat than slates.

Those in charge of hospitals and kindred institutions have for many years sought wall-covering for operating theatres, wards, or sanitary annexes, which must be of monolithic construction, impervious, and everlasting; and for occasional pavilions it is desirable, nay, more, it is even necessary that the building fabric itself should be of such a nature that it can be easily taken down and re-erected on a different site.

### An Architect's Experiments.

An architect has for some years been experimenting with different glasses, endeavouring to evolve a monolithic wall-covering, but his experiments only served to convince him of the difficulties which prevented such a material being put on the market at a reasonable price. To the architects of to-day is being submitted for consideration a material which would seem at first sight to possess many virtues, such as non-conductivity, of monolithic construction in dimensions up to 15 feet by 9 feet, varying from ½ inch to 1½ inch in thickness, and of a non-absorbent nature.

The secret of its manufacture was discovered by Mr. W. H. Turner, who represents the fourth generation of one family of Staffordshire potters, and, like many other useful inventions, it was found accidentally when he was engaged on a problem in the manufacture of ceramic ware.

This new wall-lining is glazed on both sides and edges, the joints, where found necessary, being of the type known by architects as a flush-lapped joint. Tests have been conducted to see how the material will act under severe strain, and collapse occurred at over 1,100 lb. to the inch on the ½ inch thick sample, while 140 tons of compressive test to the inch discovered failure. Frost and heat tests were applied also, and under these the samples behaved well; the former did not cause craze (hair cracks on the surface), and the latter can be appreciated when it is realised that the temperature necessary for the correct manufacture of the slabs approximates to 2000° Fahr., and that for six weeks in the kiln.

The processes of mixing and manufacture we are not fully cognisant of, but doubtless this will become public property when the necessary patents are declared.

### The Illustrations Explained.

Our illustrations, figs. 1 and 2, show the use of these slabs combined with sheradised steel in the erection of tropical buildings. That any scheme of permanent decoration is possible will be seen on examination of the interior illustration.

Simplicity of construction is an essential for the tropical building, and here we have for the erection of external walls no tool necessary except a spanner for bolting up the steelwork into which the porcelain plates are slotted and bedded in asbestos tape.

Simplicity is the keynote of construction so far as the tropical building is concerned. What is the keynote of its use to the hospital expert? Is it not monolithic construction combined with an impervious surface and no maintenance costs?

To the sanatarian it brings walls absolutely vermin-proof. Sample slabs may be seen at several places, in-

Fig. 2.—Interior of a Plate Porcelain House.

cluding the Army and Navy Stores in Victoria Street, S.W. The cost is about half-a-sovereign a yard.

These few points will serve to give an idea of what "Plate Porcelain" is. Building legislation, as at present constituted, will for some time be an obstacle to its general use, but it will overcome that in time. What do architects think of it? We invite correspondence.

## Figures and Tables

**Fig. 1. f1:**
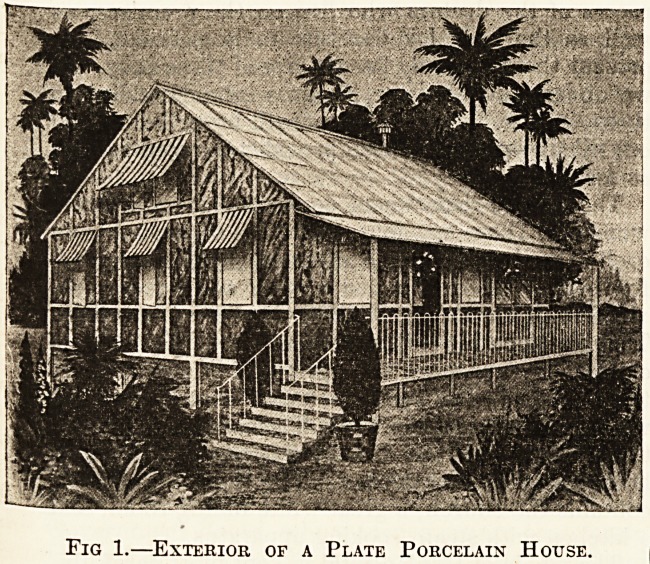


**Fig. 2. f2:**